# Rapid Immunochromatographic Detection of Serum Anti-α-Galactosidase A Antibodies in Fabry Patients after Enzyme Replacement Therapy

**DOI:** 10.1371/journal.pone.0128351

**Published:** 2015-06-17

**Authors:** Sachie Nakano, Takahiro Tsukimura, Tadayasu Togawa, Toya Ohashi, Masahisa Kobayashi, Katsuyoshi Takayama, Yukuharu Kobayashi, Hiroshi Abiko, Masatsugu Satou, Tohru Nakahata, David G. Warnock, Hitoshi Sakuraba, Futoshi Shibasaki

**Affiliations:** 1 Department of Molecular Medical Research, Tokyo Metropolitan Institute of Medical Science, 2-1-6 Kamikitazawa, Setagaya-ku, Tokyo156-8506, Japan; 2 Synthera Technologies Co., Ltd., 4-5-1 Honkomagome, Bunkyo-ku, Tokyo 113-0021, Japan; 3 Department of Functional Bioanalysis, Meiji Pharmaceutical University, 2-522-1 Noshio, Kiyose, Tokyo 204-8588, Japan; 4 Department of Gene Therapy, Institute of DNA Medicine, The Jikei University School of Medicine, 3-25-8 Nishi-Shimbashi, Minato-ku, Tokyo, 105-8461, Japan; 5 Department of Pediatrics, The Jikei University School of Medicine, 3-25-8 Nishi-Shinbashi, Minato-ku, Tokyo, 105-8461, Japan; 6 ADTEC Co., Ltd. 1693-6 Yokkaichi, Usa-City, Oita, 879-0471, Japan; 7 Nihonkai General Hospital, 30 Akiho, Sakata, Yamagata 998-8501, Japan; 8 Koujin-kai Kimachi Hospital, 1-7-13 Kimachi-dori, Aoba-ku, Sendai, Miyagi 980-0801, Japan; 9 Mutsu General Hospital, 1-2-8 Ogawa-Cho, Mutsu, Aomori 035-8601, Japan; 10 Division of Nephrology, The University of Alabama School of Medicine, 619S 19^th^ Street, Birmingham, AL 35249, United States of America; 11 Department of Clinical Genetics, Meiji Pharmaceutical University, 2-522-1 Noshio, Kiyose, Tokyo 204-8588, Japan; University of Würzburg, GERMANY

## Abstract

We developed an immunochromatography-based assay for detecting antibodies against recombinant α-galactosidase A proteins in serum. The evaluation of 29 serum samples from Fabry patients, who had received enzyme replacement therapy with agalsidase alpha and/or agalsidase beta, was performed by means of this assay method, and the results clearly revealed that the patients exhibited the same level of antibodies against both agalsidase alpha and agalsidase beta, regardless of the species of recombinant α-galactosidase A used for enzyme replacement therapy. A conventional enzyme-linked immunosorbent assay supported the results. Considering these, enzyme replacement therapy with agalsidase alpha or agalsidase beta would generate antibodies against the common epitopes in both agalsidase alpha and agalsidase beta. Most of the patients who showed immunopositive reaction exhibited classic Fabry phenotype and harbored gene mutations affecting biosynthesis of α-galactosidase A. As immunochromatography is a handy and simple assay system which can be available at bedside, this assay method would be extremely useful for quick evaluation or first screening of serum antibodies against agalsidase alpha or agalsidase beta in Fabry disease with enzyme replacement therapy.

## Introduction

α-Galactosidase A (GLA, EC 3. 2. 1. 22) is a lysosomal hydrolase encoded by a *GLA* gene localized at Xq22, and it catalyzes the degradation of glycolipids, predominantly globotriaosylceramide (Gb3) and globotriaosylsphingosine (lyso-Gb3). The mature form of GLA is a glycoprotein consisting of 398 amino acids and sugar chains, and the native GLA from humans has a homodimeric structure [1].

Deficient activity of GLA causes systemic accumulation of the glycolipids, leading to Fabry disease (MIM 301500). The manifestations in the “classic form” of Fabry males involve acroparesthesia, angiokeratomas, hypohidrosis and corneal opacities during childhood or adolescence, and develop kidney, heart and cerebrovascular involvement in adulthood. On the other hand, Fabry males with the “later-onset form” develop heart and/or kidney disease without the childhood symptoms. Fabry females exhibit a wide range of clinical presentations due to random X-chromosomal inactivation [1,2].

Two different recombinant GLAs produced in human fibroblasts (agalsidase alpha, Aga-A; Replagal, Shire Human Genetic Therapies) [3] and Chinese hamster ovary cells (agalsidase beta, Aga-B; Fabrazyme, Genzyme) [4,5] are available for enzyme replacement therapy (ERT) for Fabry disease. The ERT improves the clinical manifestations or prevents the progression of the disease, if the treatment is started early, and many Fabry patients have been successfully treated with these recombinant GLAs [6,7]. However, recurrent injections of the recombinant GLAs often cause the production of antibodies against them among Fabry male patients, leading to allergic reactions and/or reduction of the efficacy of ERT [8–10]. Many studies involving detection of anti-GLA antibodies have been performed for each recombinant GLA by means of enzyme-linked immunosorbent assay (ELISA) method [11,12]. However, ELISA is not suitable for quick evaluation at bedside. Furthermore, there are methodological differences among studies, *i*.*e*., sample treatment, definitions of seropositivity, and cut-off points [8], and the results have often made a confusion in comparing the immune reaction due to each recombinant GLA. An effort has been directed to standardize GLA antibody assays across the industry in order to obtain comparative antibody data on ERT [13], but it seems that the standardization does not successfully work.

In this study, we developed an easy and rapid immunochromatography (IC)-based assay method for detecting antibodies against the recombinant GLAs, and measured anti-GLA antibody levels in serum from Fabry patients treated with Aga-A, Aga-B, or the both as samples. Furthermore, we validated this IC method and compared the results with those of the conventional ELISA.

## Materials and Methods

This study was approved by the Ethics Committees of Tokyo Metropolitan Institute of Medical Science, Meiji Pharmaceutical University, Jikei University School of Medicine, The University of Alabama School of Medicine, Nihonkai General Hospital, Oujin-kai Kimachi Hospital, Mutsu General Hospital. All participants and/or their parents provided written informed consent to participate in this study.

### Reagents

Aga-A is a gift from Shire Human Genetic Therapeutics (Cambridge, MA, USA), and Aga-B was purchased from Genzyme Japan (Tokyo, Japan). SureBlue Reserves TMB microwell peroxidase substrate (1-Component) and TMB Stop Solution were purchased from Kirkegaard & Perry Laboratories, Inc. (Gaithersburg, MD, USA). Calf-intestinal alkaline phosphatase (AP), and the substrate, nitro-blue tetrazolium and 5-bromo-4-chloro-3'-indolyphosphate (NBT/BCIP) were purchased from Thermo Fisher Scientific Inc. (Rockford, IL, USA). ZyMAX AP- or HRP-labelled goat anti-human IgG (H+L) were from Life technologies (Camarillo, CA, USA). Recombinant human acid α-glucosidase, α-L-iduronidase, and iduronate-2-sulphatase were purchased from Genzyme Japan.

### Patients and samples

Serum samples for measurement of the antibodies against Aga-A and Aga-B were obtained from 26 Fabry males (17 classic type, 5 later-onset type, and 4 unknown), 3 Fabry females (Table 1), and control as an average from 20 healthy volunteers (S1 Table). The clinical information of the patients (age, phenotype, genotype, and recombinant GLA with which a patient was treated on ERT) was shown in Table 1. Phenotype of the Fabry patients was classified based on the clinical manifestations, family history and results of *GLA* gene analysis, and Fabry gene mutations were classified according to the Fabry database (http://fabry-database.org/). Among the Fabry patients, 13 ones have been treated with Aga-A, 13 ones with Aga-B, and 3 ones with the both for more than 3 months (Table 1).

**Table 1:** Measurement of serum anti-GLA antibodies in Fabry patients received ERT and their phenotype and genotype.

				ELISA						IC^3)^		
No.	Phenotype	Age	Genotype	Ag: Aga-A^1)^		Ag: Aga-B^2)^		Ag: (-)		Ag: Aga-A	Ag: Aga-B	ERT^6)^
				OD^4)^	SD	OD	SD	OD	SD	Score^5)^	Score	
1	Classic Fabry male	18	W47X	3.064	0.070	2.936	0.146	0.028	0.003	8	8	Aga-A
2	Fabry male	NA^7)^	NA^7)^	2.738	0.068	2.924	0.147	0.035	0.002	8	8	Aga-A
3	Fabry male	47	NA^7)^	2.217	0.032	2.192	0.031	0.012	0.004	7	8	Aga-A
4	Fabry male	24	NA^7)^	0.159	0.004	0.166	0.002	0.059	0.001	1	1	Aga-A
5	Fabry female	9	Y365X/WT^8)^	0.171	0.009	0.179	0.014	0.244	0.007	0	0	Aga-A
6	Fabry male	23	NA^7)^	0.054	0.005	0.064	0.001	0.069	0.014	0	0	Aga-A
7	Later-onset Fabry male	15	R112H	0.039	0.001	0.037	0.002	0.047	0.003	0	0	Aga-A
8	Later-onset Fabry male	11	M296I	0.027	0.007	0.044	0.005	0.057	0.008	0	0	Aga-A
9	Classic Fabry male	21	R227X	0.021	0.002	0.032	0.001	0.020	0.001	0	0	Aga-A
10	Later-onset Fabry male	61	N215S	0.014	0.001	0.031	0.001	0.050	0.002	0	0	Aga-A
11	Classic Fabry male	59	IVS5-1G>C	0.014	0.004	0.017	0.002	0.037	0.001	0	0	Aga-A
12	Later-onset Fabry male	59	M296I	0.012	0.001	0.024	0.004	0.002	0.003	0	0	Aga-A
13	Fabry female	57	NA^7)^	0.009	0.002	0.012	0.002	0.015	0.003	0	0	Aga-A
14	Classic Fabry male	44	R227X	3.087	0.252	3.015	0.014	0.04	0.005	8	8	Aga-B
15	Classic Fabry male	51	W277X	3.016	0.052	2.777	0.124	0.034	0.003	7	8	Aga-B
16	Classic Fabry male	47	W340X	2.742	0.087	2.740	0.019	0.017	0.002	8	8	Aga-B
17	Classic Fabry male	48	c.717_718delAA	0.740	0.009	0.808	0.017	0.036	0.002	3	5	Aga-B
18	Classic Fabry male	55	c.1118delG	0.055	0.001	0.081	0.003	0.013	0.005	0	1	Aga-B
19	Classic Fabry male	35	L120P, A121T	0.052	0.001	0.065	0.003	0.236	0.004	0	1	Aga-B
20	Classic Fabry male	52	W277X	0.047	0.002	0.051	0.002	0.019	0.003	0	0	Aga-B
21	Classic Fabry male	42	D264Y, V269M	0.030	0.002	0.047	0.008	0.028	0.006	0	0	Aga-B
22	Classic Fabry male	52	N272S	0.031	0.003	0.035	0.002	0.037	0.003	0	0	Aga-B
23	Classic Fabry male	41	N272K	0.024	0.002	0.034	0.004	0.014	0.004	0	0	Aga-B
24	Classic Fabry male	23	R227Q	0.024	0.003	0.030	0.002	0.047	0.005	0	0	Aga-B
25	Classic Fabry male	37	R112S	0.020	0.002	0.021	0.001	0.029	0.002	0	0	Aga-B
26	Fabry female	NA	NA^7)^	0.014	0.001	0.026	0.013	0.028	0.005	0	0	Aga-B
27	Classic Fabry male	44	Large Deletion	2.897	0.134	2.817	0.082	0.028	0.008	7	8	Aga-A Aga-B
28	Later-onset Fabry male	59	L403S	0.486	0.008	0.527	0.016	0.013	0.002	2	5	Aga-A Aga-B
29	Classic Fabry male	51	IVS6+1G>T	0.029	0.002	0.027	0.011	0.01	0.001	0	0	Aga-A Aga-B
Cont^8)^				0.035	0.023	0.038	0.026	0.047	0.051	0	0	-

^1)^Aga-A; agalsidase alpha

^2)^Aga-B; agalsidase beta

^3)^IC; immunochromatography

^4)^OD; optical density at 450nm

^5)^Score: 8 scale-values of line density by visual measurement

^6)^ERT; enzyme replacement therapy

^7)^NA; not available

^8)^WT: wild type. Each serum was diluted 100 folds and 5 folds with healthy control serum before ELISA and IC, respectively.

^8)^Cont: average values of healthy control (Refer to S1 Table).

### Immunochromatography (IC)

**Fig 1:**
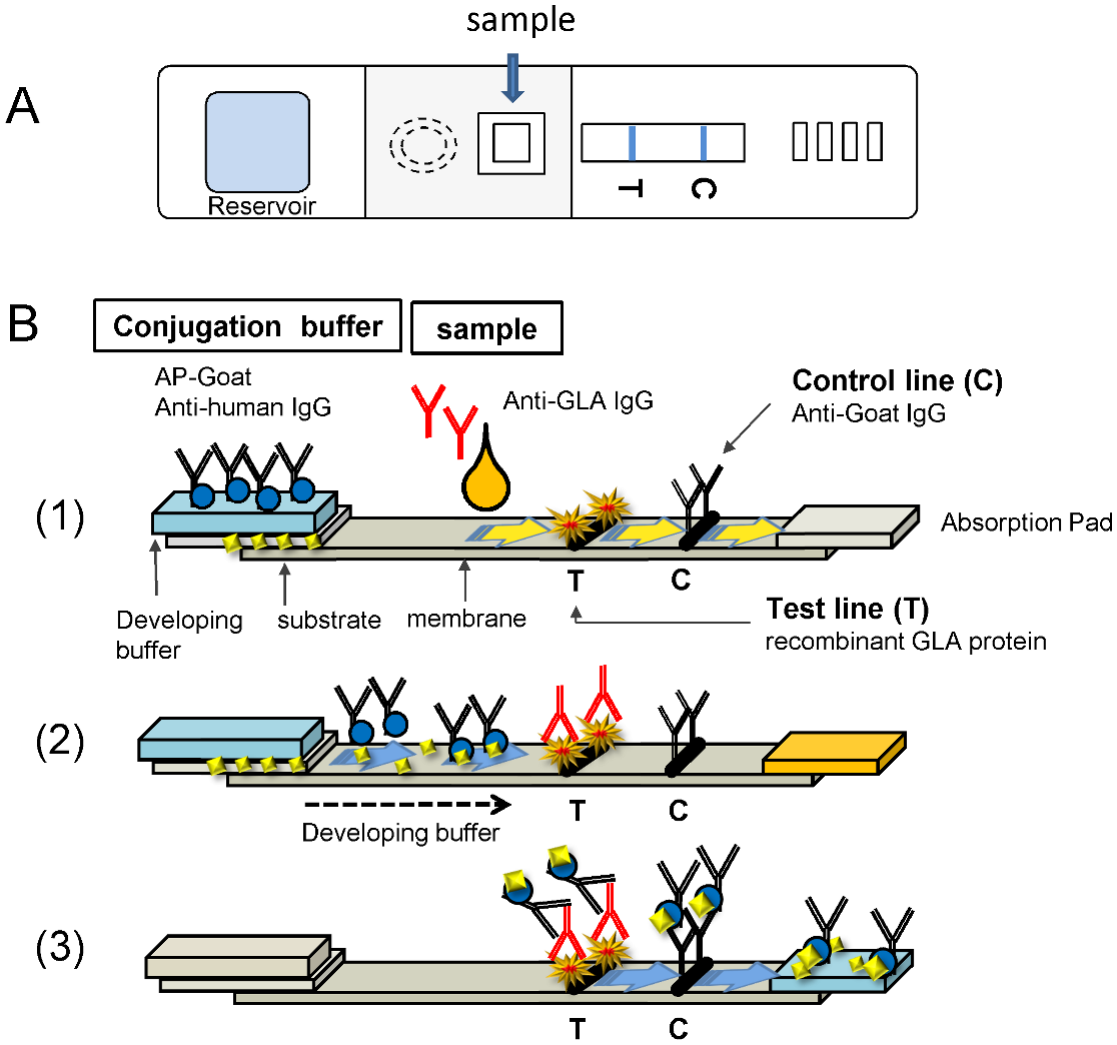
Scheme of IC for detection of anti-GLA antibodies in serum. A. Scheme of an IC chip which was designated for detecting anti-GLA antibodies in serum. Sample is dropped into the middle hole (arrow) and the reaction buffer including AP-labelled anti-human IgG in the reservoir unit is running on the membrane after a break by finger. C means control line and T does test line. B (1). The principle of IC for Aga-A and Aga-B. Mixture solution of serum and buffer including anti-GLA antibodies from serum of Fabry patients who received ERT with Aga-A and/or Aga-B was applied into the sample hole, and then the solution is running in the nitrocellulose membrane. B (2). After breaking the reservoir unit (Reservoir), the reaction buffer including AP-labelled anti-human IgG and substrate for AP which was stored in separate space in the reservoir unit is running on the membrane. B (3). If the sample has anti-GLA antibodies, the detection antibody on the membrane of IC captures the antibody/AP-labelled anti-human antibody/AP-substrate complex, and blue line appears on the test line (T). On the control line. The AP-labelled anti-human IgG antibody is trapped and makes blue color line, showing that the system works well (C). B. The same samples were applied to IC for Aga-A (upper lane) and Aga-B (lower lane), and the color line was scored from 0 to 8 after 15 min later. The color scale is shown in C.

For detecting anti-recombinant GLA (Aga-A or Aga-B) antibodies in serum, Aga-A or Aga-B (1 μg/line) were immobilized on the IC membrane (Fig 1, Test line; T). In contrast, anti-goat IgG antibody was immobilized on the control line (Fig 1, Control line: C) to evaluate the appropriate flow of IC by detecting goat anti-human IgG as shown in Fig 1A. In the first step, serum, which was diluted at 10 folds with sample buffer (50 mM Tris-HCl (pH = 7.2), 150 mM NaCl, 1% Trition X-100), was dropped on the IC chip (Fig 1B(1)). In the second step, the reservoir unit (Reservoir) containing conjugation buffer (50 mM Tris-HCl (pH = 7.2), 150 mM NaCl, 1% Trition X-100, 1 mM MgCl_2_, and AP labelled-goat anti-human IgG) was opened by fingers for developing the antibody/antigen reaction (Fig 1B(2)). The dry substrate of AP (BCIP) was fixed on the membrane and mixed with conjugation buffer after opening the Reservoir. In the final step, anti-GLA antibodies captured by the immobilized Aga-A or Aga-B on the membrane were detected by alkaline phosphatase (AP)-conjugated goat anti-human IgG, and visualized with enzyme reaction of AP (Fig 1B(3)). The level of the color strength (score) was evaluated from level 0 (no color) to level 8 (maximum density) by the visual determination according to a control color paper. We determined here that the immune reaction was positive (+) when the score was 2 or more, and pseudopositive (±) when it was one.

To examine the cross-reactivities of the recombinant GLAs and other lysosomal enzymes including acid α-glucosidase, α-L-iduronidase, and iduronate-2-sulfatase to the antibodies, we prepared IC kits with membranes on which each enzyme (1 μg/strip) was immobilized, and the immune reaction was examined using serum from Fabry patients as samples according to the same procedure of the GLAs.

### Magnetic beads ELISA

For the magnetic beads ELISA, Aga-A or Aga-B were each conjugated to Tosyl-activated Dynabeads M-280 (Invitrogen Dynal AS, Oslo, Norway) according to the manufacturer’s protocol. The 96-well plate wells were blocked by incubating with 200 μL/well aliquot of Pierce Protein-Free Blocking Buffer (Thermo Fisher Scientific K.K. Pierce Biotechnology, Rockford, IL, USA) for 1 h at room temperature. Then the blocking buffer was removed. The recombinant GLA protein-conjugated magnetic beads were mixed in an assay buffer containing 0.05% Tween-20, 0.45 M NaCl, 50 mM sodium phosphate, pH 7.4, and 10% goat serum. In the next, they were mixed with serum, which were appropriately diluted with 0.05% Tween-20, 0.45 M NaCl, 50 mM sodium phosphate, pH 7.4, in a 96-well plate for 1 h at room temperature with shaking. The beads were captured for 3 min with a 96-well Magnetic-Ring Stand (Applied Biosystems, Foster City, CA), and washed 4 times with 180 μL/well aliquot of wash buffer (0.05% Tween-20, 0.5 M NaCl, and 20 mM Tris-HCl, pH7.4). Then, an appropriately diluted HRP-conjugated Goat anti-Human IgG (H+L) antibody was added to the test wells at 50 μL/well, and the 96-well plate was incubated for 1 h at room temperature. The conjugate solutions were removed and the wells were washed four times. Finally, the immune reaction was analyzed using the microplate reader Benchmark (BIO-RAD, Hercules, CA, USA) by adding peroxidase substrate (0.04% o-phenylenediamine, 0.6% H_2_O_2_, and 0.15 M citric acid buffer, pH 5.0) to each well.

### Statistical analyses

Student’s *t* test was performed using Excel 2013 (Microsoft, Redmond, WA) to determine the difference between the classic group and other ones. Data are expressed as means ± standard deviation (SD). To examine the distribution of the cases in each phenotypic group, dot plot analysis (http://onlinestabook.com/2/graphing-distribution/boxplot.html) was performed.

## Results

### Establishment of IC for measurement of serum anti-GLA antibody level

**Fig 2:**
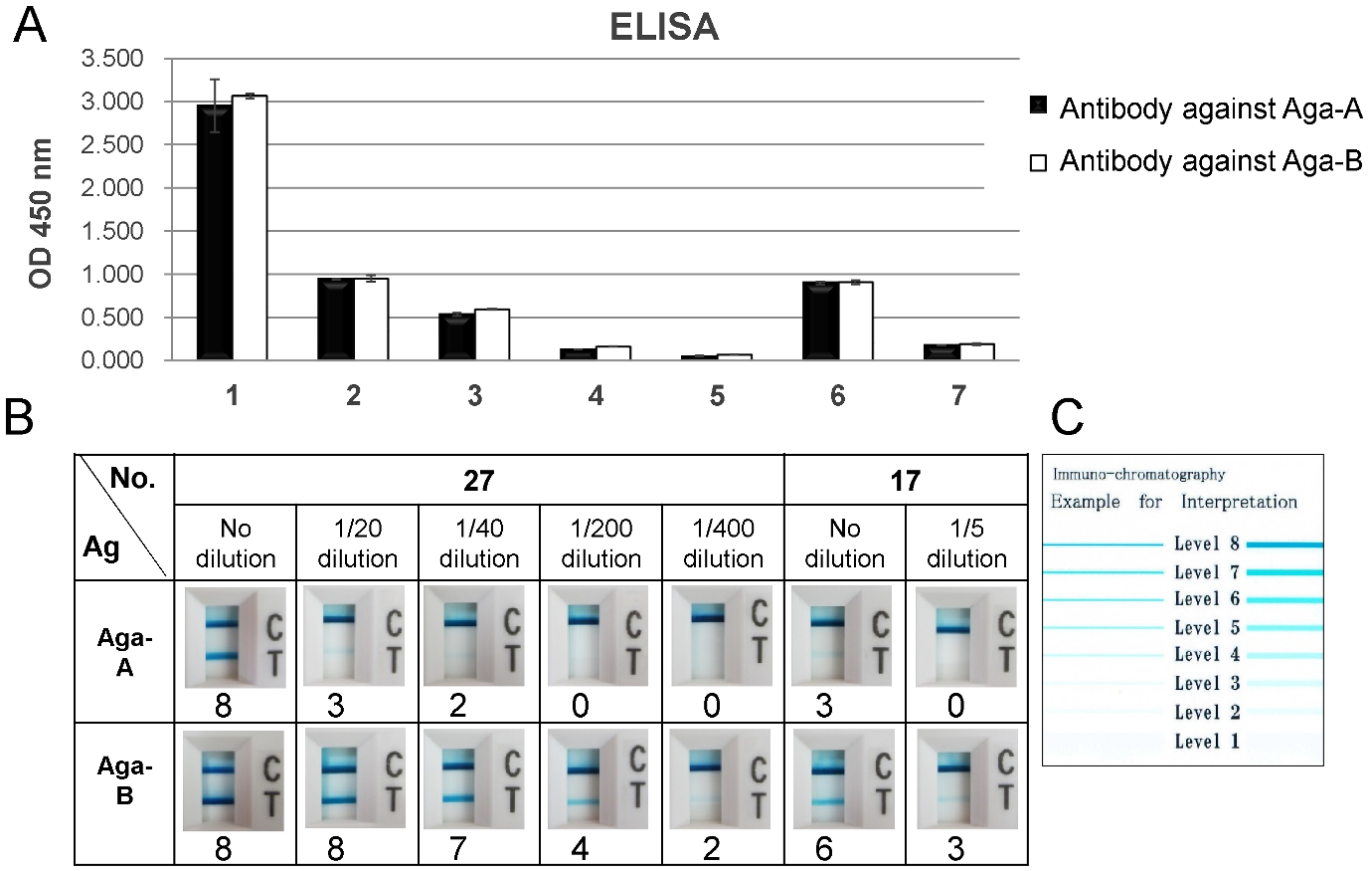
Characterization of ELISA and IC. A. The serum of samples #27 and #17 were assayed by ELISA for antibodies against Aga-A (black column) and Aga-B (white column) as shown in lanes 1 and 6, and also assayed after dilution of 1/20 (lane 2), 1/40 (lane 3), 1/200 (lane 4), and 1/400 (lane 5) for #27, and 1/5 (lane 7) for #17, respectively, with 0.05% Tween-20, 0.45M NaCl, 50mM sodium phosphate, pH7.4. HRP-labelled anti-human IgG antibody was applied and then washed out. The values of OD 450nm were measured after applying the HRP substrate. The data were shown as mean ± SD. B. The same samples were applied to IC for Aga-A (upper lane) and Aga-B (lower lane), and the color line was scored from 0 to 8 after 15 min later. The color scale is shown in **C**.

For the first evaluation of the developed ICs, we used two serum samples which had exhibited high titer of anti-GLA antibodies in the previous study; the sample #17 from a patient who had received ERT with Aga-A and #27 from one with both Aga-A and Aga-B for comparison with the results of authentic ELISA (Fig 2). We measured anti-Aga-A antibodies (black column) and anti-Aga-B antibodies (white column) for the samples at the indicated dilution by means of the ELISA (Fig 2A). In this experiment, the original serum were diluted with normal serum and used as samples. The results clearly showed the correlation to the dilution rate without background. Next, we applied the same samples to two kinds of ICs; one plotted with Aga-A (upper lane) and another with Aga-B (lower lane) (Fig 2B), and we scored each test by visual judgment according to the color scale paper (Fig 2C). Both samples without dilution clearly showed an immune reaction against both Aga-A and Aga-B. The strength of test line (T) was well correlated to the dilution rates. The sample #27 from a patient who had received ERT with Aga-A clearly indicated the cross-reaction to Aga-B.

### Specificity of the developed IC

Next, we tested the cross reaction to other similar enzymes related to lysosomal storage diseases such as Pompe disease (acid α-glucosidase deficiency), mucopolysaccharidosis I (α-L-iduronidase deficiency), and mucopolysaccharidosis II (iduronate-2-sulphatase deficiency). The samples #27, 28, and 29 were assayed to examine the cross reaction to acid α-glucosidase, α-L-iduronidase, and iduronate-2-sulfatase with ICs which were designated to detect serum antibodies against each enzyme. The samples #27 and 28 with high scores of antibodies against both Aga-A and Aga-B demonstrated no immune reaction against the three enzymes. The sample #29, which exhibited score 0 in ICs for both Aga-A and Aga-B, did not show any reaction against the three enzymes (S2 Table).

### Comparison of IC versus ELISA as to serum from Fabry patients and healthy controls

To validate IC and ELISA using clinical samples, the levels of anti-GLA antibodies in serum from Fabry patients treated with Aga-A, Aga-B, or the both were measured. First, we performed ELISA and IC assay using serum from 20 healthy subjects, and evaluated the control values (S1 Table). The OD 450nm values of both ELISAs using Aga-A and Aga-B were in the range from 0.008 to 0.078 (Aga-A) and 0.008 to 0.094 (Aga-B). On the other hand, both ICs for Aga-A and Aga-B showed score 0 for all the healthy subjects.

In total 29 Fabry patients who received ERT, three patients treated with Aga-A (#1–3), 4 ones with Aga-B (#14–17), and 2 ones with both Aga-A and Aga-B (#27, 28) were immunopositive for the ICs (S1 Fig), scored by a color scale. They are all male patients, and their clinical phenotypes are 6 classic, 1 later-onset, and 2 unknown. Gene analyses were performed for seven patients of them, and the results revealed that these patients harbored deletions (c.717_718delAA and a large deletion), nonsense mutations (W47X, R227X, W277X, and W340X), and a missense one (L403S). Beside them, one male patient who had received ERT with Aga-A (#4: phenotype unknown, genotype not examined) exhibited score 1 (pseudopositive) for both Aga-A and Aga-B, and two male patients treated with Aga-B (#18: phenotype classic, genotype c.1118delG; and #19: phenotype classic, genotype L120P and A121T) exhibited score 1 for Aga-B, but not for Aga-A. Eight classic Fabry males, five later-onset Fabry males, one Fabry male of unknown phenotype, and three Fabry females were immunonegative for ICs. Gene analyses were performed for 14 patients of them, and the results showed that these patients harbored nonsense mutations (R227X, W277X, and Y365X), splicing defects (IVS5-1G>C and IVS6+1G>T), and missense mutations (R112H, R112S, N215S, R227Q, D264Y+V296M, N272S, N272K, and M296I x 2) (Table 1).

**Fig 3:**
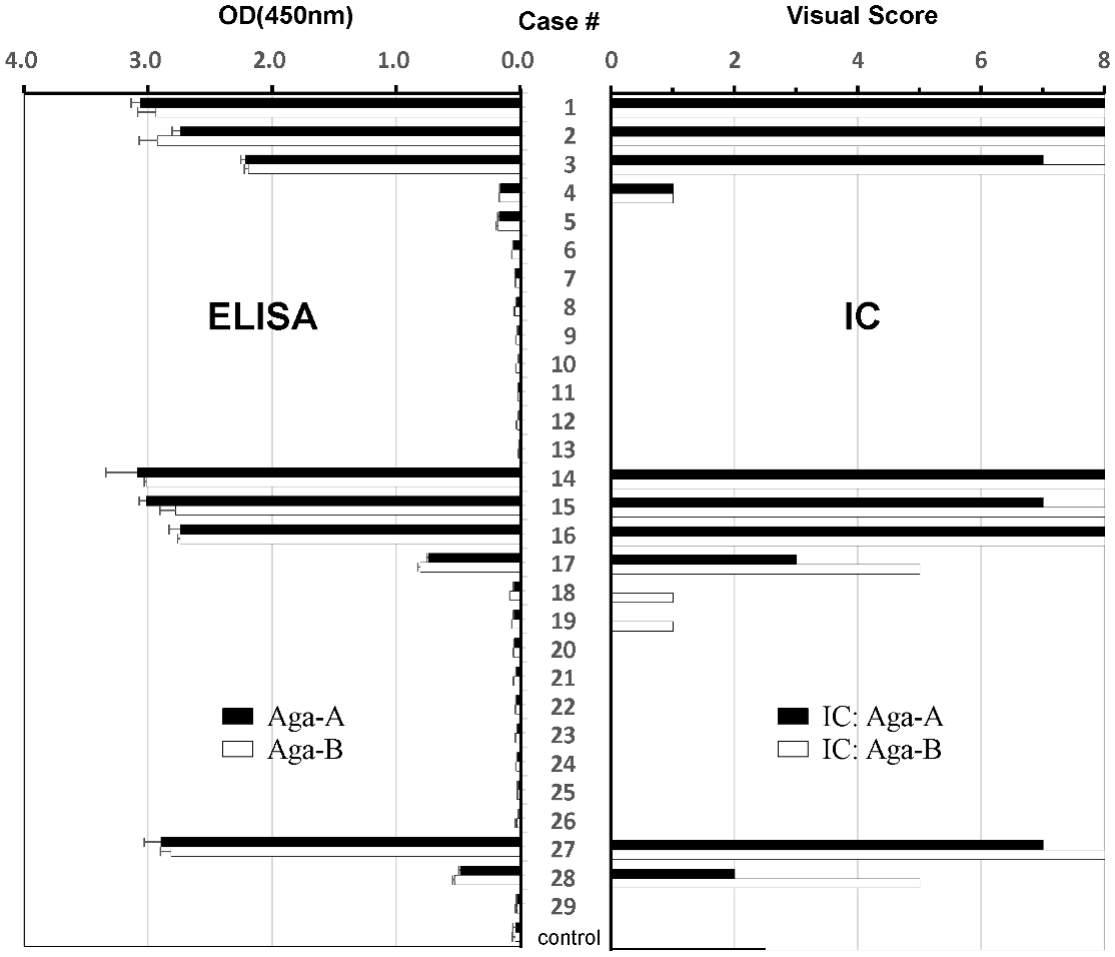
Comparison of anti-GLA antibody levels in clinical samples using ELISA and IC. Sera from 29 Fabry patients (#1–29) and controls (Cont) were assayed by ELISA (left side) and IC (right side) as refer to Table 1. Control exhibits the average value of the data of healthy people (n = 20) used blank ELISA without both Aga-A neither Aga-B. According to the protocol, ELISA was performed using magnetic beads attached with Aga-A or Aga-B as antigens and the anti-GLA antibodies in each 100-fold diluted serum were measured with colorimetric methods of OD 450 nm using AP-labelled-anti-human IgG antibody and the substrate. The same serum were 5-fold diluted and applied to IC for Aga-A (black column) and Aga-B (white column), and judged by the color scale. The all data were shown as mean ± SD.

**Fig 4:**
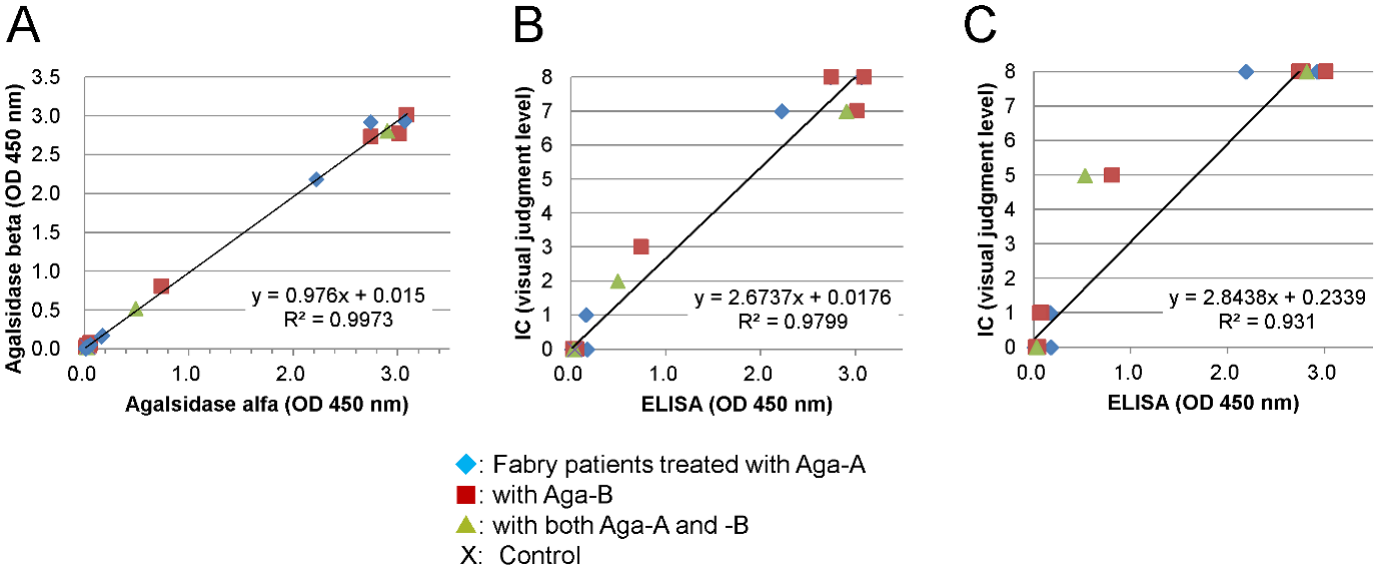
Measurement of anti-GLA antibody levels using ELISA and IC. A. The relationship between the anti- Aga-A antibody level and anti-Aga-B antibody one measured by means of ELISA. B. The relationship between IC and ELISA for anti-GLA antibody level using Aga-A. C. The relationship between IC and ELISA for anti-GLA antibody level using Aga-B. In each figure, closed rhombuses (♦), closed boxes (■), closed triangles (▲), and crosses (×) show the data for samples from Fabry patients treated with Aga-A, Aga-B, and the both, and control subjects, respectively. Control exhibits the average value of the data of healthy people (n = 20).

The results of the measurement of anti-GLA antibodies with ELISA and IC were summarized in Table 1, and the result of the comparative analysis for them was shown in Fig 3. The OD values of serum including antibodies against Aga-A (black column) and Aga-B (white column) in ELISA (left panel) were well correlated with their visual scores in IC (right panel). The results of the ELISA using Aga-A were well correlated with those using Aga-B (R^2^ = 0.9973), as shown in Fig 4A. The relationship between the scores in IC and the OD values in ELISA was also examined. The results demonstrated that the scores for IC using Aga-A were well correlated with the OD values for ELISA using Aga-A (R^2^ = 0.9799), as shown in Fig 4B. The results of IC using Aga-B were also well correlated with those for ELISA using Aga-B (R^2^ = 0.931), as shown in Fig 4C.

### Evaluation of the non-specific reaction of serum factors

Although well correlation between the OD values in the ELISAs and the scores in the IC ones was observed for most of the Fabry cases, there were exceptional cases. The sample #5 exhibited moderately high OD values (0.171 for Aga-A and 0.179 for Aga-B), but the result of the IC assay showed score 0 for both Aga-A and Aga-B. On the other hand, the samples #18 and #19 exhibited score 1 although the OD values were low (0.55 and 0.81, and 0.052 and 0.065 for Aga-A and Aga-B, respectively). To evaluate the discrepancy of these results, we prepared the ELISA with magnetic beads fixed with Aga-A, Aga-B, or without antigen (Antigen (-)), and assayed serum samples with and without pre-absorption of bovine serum albumin (BSA), or Aga-A, or Aga-B (S2, S3, S4, and S5 Figs). The sample #5 showed moderately high values of OD 450 nm (0.17–0.19) in ELISA with Aga-A (black column) as summarized in S3 Table. However, the values were higher than those in the ELISA without antigen (white column), and there was not any change after the absorption of BSA or Aga-A (S2 Fig). On the other hand, the sample #2 clearly showed high values in the ELISA with Aga-A, but not without antigen. The values are also well absorbed by the addition of Aga-A (S3 Fig). The same experiments were performed for the samples #19 (S4 Fig) and #14 (S5 Fig) as summarized in S4 Table. Interestingly, in the case of sample #19, absorption of BSA completely suppressed the back ground. These results suggest that we have to consider the addition of BSA in the assay buffer and the visual score 1 must be carefully evaluated as a border line (pseudopositive).

## Discussion

According to an increase in the number of Fabry patients treated with the recombinant GLAs, an easy and rapid detection of antibodies against them is urgently needed to properly follow the patients during ERT. IC has been used for helping various clinical diagnoses at bedside, *i*.*e*., detection of viruses [14–16]. In this study, we developed an IC-based assay method for detecting anti-GLA antibodies in serum. This assay requires only one drop volume of serum or whole blood as a sample and can rapidly measure anti-GLA antibodies.

Using this new method, we determined the levels of antibodies against Aga-A and Aga-B for Fabry patients who had been treated with Aga-A, Aga-B or the both. The results revealed that the Fabry patients exhibited the same levels of antibodies against both Aga-A and Aga-B regardless of the species of recombinant GLA used for ERT. The results of authentic ELISA supported those of the IC assay. Aga-A and Aga-B are produced in human fibroblasts and Chinese hamster ovary cell, respectively, and it has been reported that they have the same amino acid sequences, although their components of sugar chains are different according to their host cells in production [17]. Considering that Aga-A and Aga-B are indistinguishable in terms of antibody cross-reactivity, ERT with Aga-A or Aga-B would generate antibodies against the common epitopes, probably in the protein portion of the GLA molecule. There have been few reports involving measurement of titers of anti-GLA antibodies in Fabry patients treated with either Aga-A or Aga-B under the same assay condition [18–20]. Linthorst *et al*. reported that after 6 months of treatment with either Aga-A or Aga-B, some patients showed high titers of IgG antibodies that cross-react in vitro similarly with the both [18]. The result supports our data.

Among the nine male patients who showed an immunopositive reaction against the recombinant GLAs, six exhibited the classic phenotype and one later-onset, and as to the other two patients the phenotype was unknown. Among the three patients who showed a pseudopositive reaction, two exhibited the classic phenotype, as to the other one patient the phenotype was unknown. On the other hand, nine classic Fabry males, four later-onset ones and one male patient with unknown phenotype showed an immunonegative reaction against Aga-A or Aga-B. Immune reaction of the female Fabry patients was all negative.

Relationship between gene mutations and immune reaction was also examined. Two deletions, four nonsense mutations and one missense mutations were identified in the patients who showed an immunopositive reaction, and as to the other two cases gene analysis was not performed because the informed consent cannot be obtained from them. One deletion and one missense mutation were found in the patients who showed a pseudopositive reaction, and as to the other one case gene analysis was not performed. Two splicing defects, 3 nonsense mutations, and 9 missense ones were identified in the patients who showed an immunonegative reaction, and as to the other three cases gene analysis was not performed (Table 1).

These results revealed that six of the nine immunopositive cases exhibited the classic phenotype, and their genotypes were deletions (2 cases) or nonsense mutations (4 cases), which lead to defect of biosynthesis of GLA. It suggests that loss of GLA protein accelerates generation of anti-GLA antibodies during ERT. Previously, we reported that most of the classic type of Fabry males exhibit no enzyme activity and no or trace of GLA proteins in plasma, although the later-onset type of Fabry males had residual enzyme activity and small or considerable amount of GLA proteins [21]. It suggests that classic Fabry patients tend to generate anti-GLA antibodies rather than the later-onset ones.

In conclusion, we developed an easy and rapid IC-based assay for detecting anti-GLA antibodies in serum. It will be useful for quick evaluation or first screening of serum antibodies against Aga-A or Aga-B. Our IC-based detection system of anti-GLA antibodies may also help to follow the height of the titer in an individual treated with ERT by means of a score card.

## Supporting Information

S1 Fig Immunochromatographic detection of antibodies against Aga-A and Aga-B. The serum were 5-fold diluted and applied to IC for Aga-A (upper panel) or Aga-B (lower panel). The color scale was shown in the right panel.
(TIF)

S2 Fig Evaluation of the non-specific reaction of serum factors in the sample #5. To evaluate discrepancy of the results of ELISA and IC in the sample #5, we assayed the serum with or without pre-absorption of 1% BSA or 10 μg of Aga-A or Aga-B in ELISA fixed with (black column) or without Aga-A (white column) as an antigen.
(TIF)

S3 Fig Positive control using the serum from sample #2. (TIF)

S4 Fig Evaluation of the non-specific reaction of serum factors in the sample #19. We assayed the serum #19 with or without pre-absorption of 1% BSA or 10 μg of Aga-A or Aga-B in ELISA fixed with (black column) or without Aga-A (white column) as an antigen.
(TIF)

S5 Fig Positive control using the serum from sample #14. (TIF)

S1 Table The average values of Anti GLA Antibody in health control. (DOCX)

S2 Table Specificity of Aga-A IC or Aga-B IC for serum or plasma anti-GLAs antibodies in Fabry Patients. The average values of anti-GLA antibodies in healthy controls.
(DOCX)

S3 Table Specificity of ELISA with or without pre-absorption of BSA and/or Aga-A in samples from #2 and #5. (DOCX)

S4 Table Specificity of ELISA with or without pre-absorption of BSA and/or Aga-B in samples from #14 and #19. (DOCX)
