# Supplementary figures and images for: Rapid Immunochromatographic Detection of Serum Anti-α-Galactosidase A Antibodies in Fabry Patients after Enzyme Replacement Therapy
Source: PLoS One. 2015 Jun 17;10(6):e0128351. doi: 10.1371/journal.pone.0128351 (PMC4470989; doi:10.1371/journal.pone.0128351)

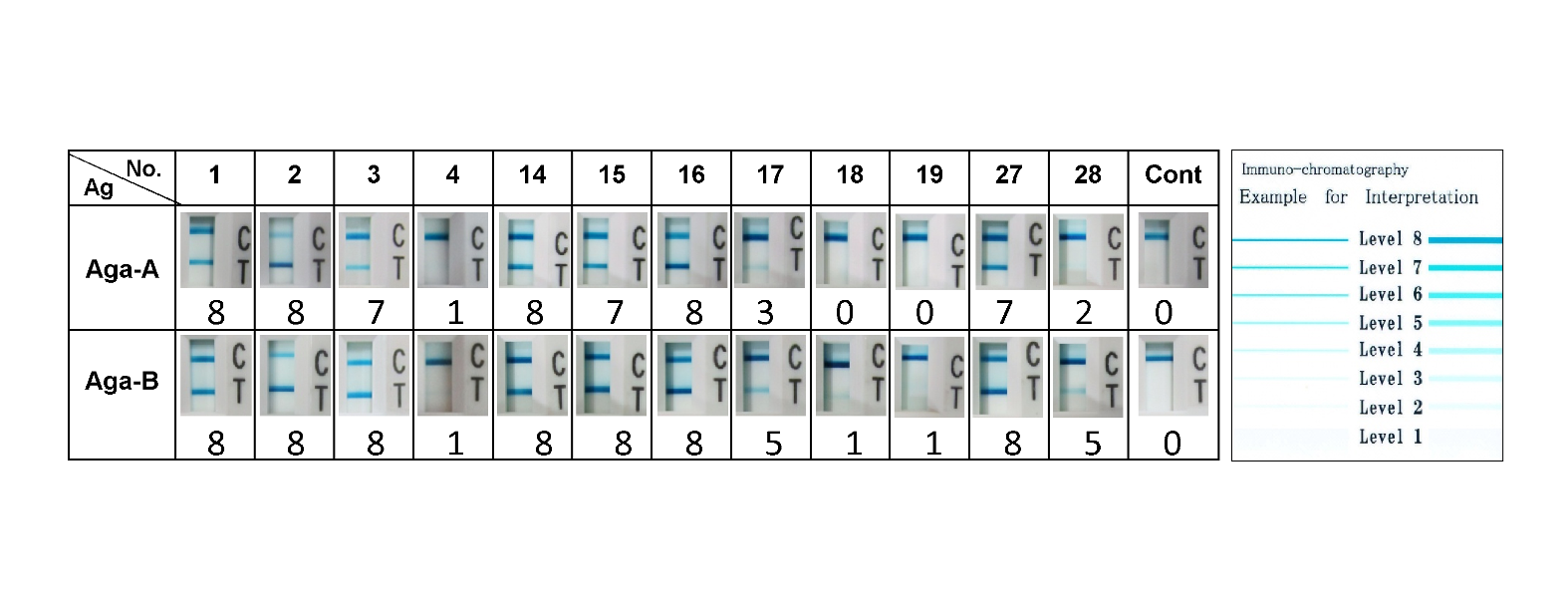

Supplement: S1 Fig — The serum were 5-fold diluted and applied to IC for Aga-A (upper panel) or Aga-B (lower panel). The color scale was shown in the right panel. (TIF) [file pone.0128351.s001.tif]

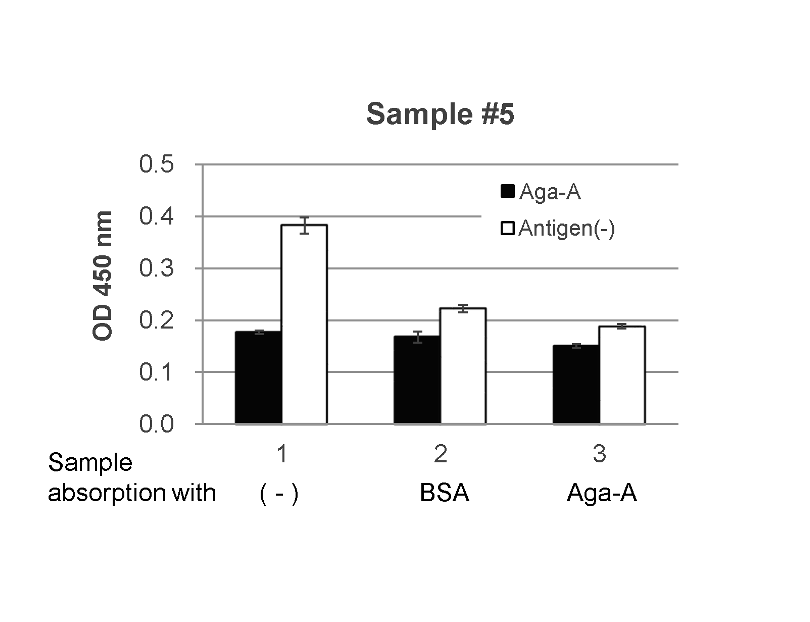

Supplement: S2 Fig — To evaluate discrepancy of the results of ELISA and IC in the sample #5, we assayed the serum with or without pre-absorption of 1% BSA or 10 μg of Aga-A or Aga-B in ELISA fixed with (black column) or without Aga-A (white column) as an antigen. (TIF) [file pone.0128351.s002.tif]

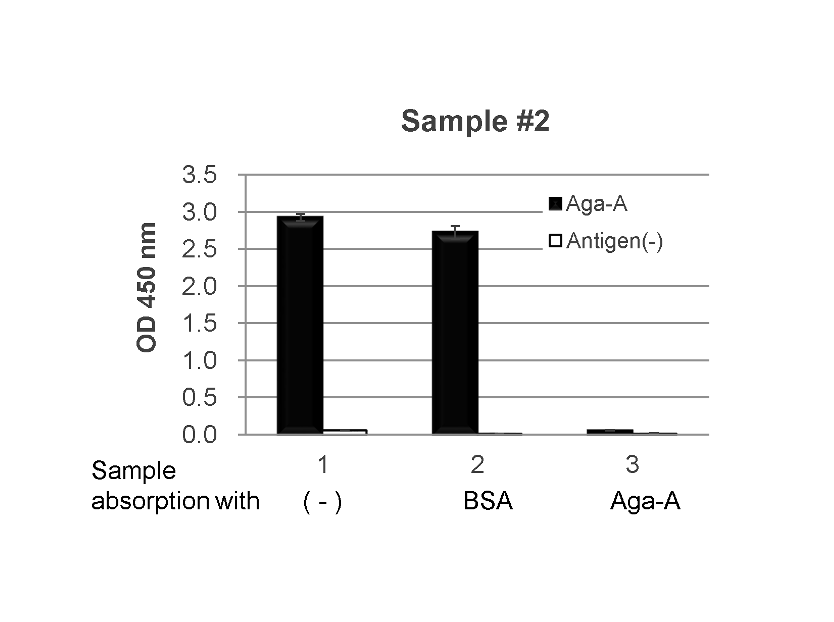

Supplement: S3 Fig — (TIF) [file pone.0128351.s003.tif]

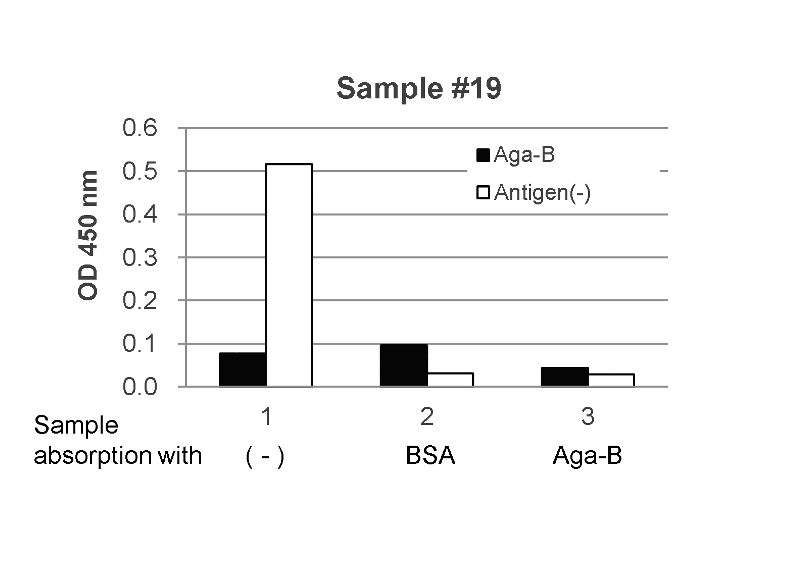

Supplement: S4 Fig — We assayed the serum #19 with or without pre-absorption of 1% BSA or 10 μg of Aga-A or Aga-B in ELISA fixed with (black column) or without Aga-A (white column) as an antigen. (TIF) [file pone.0128351.s004.tif]

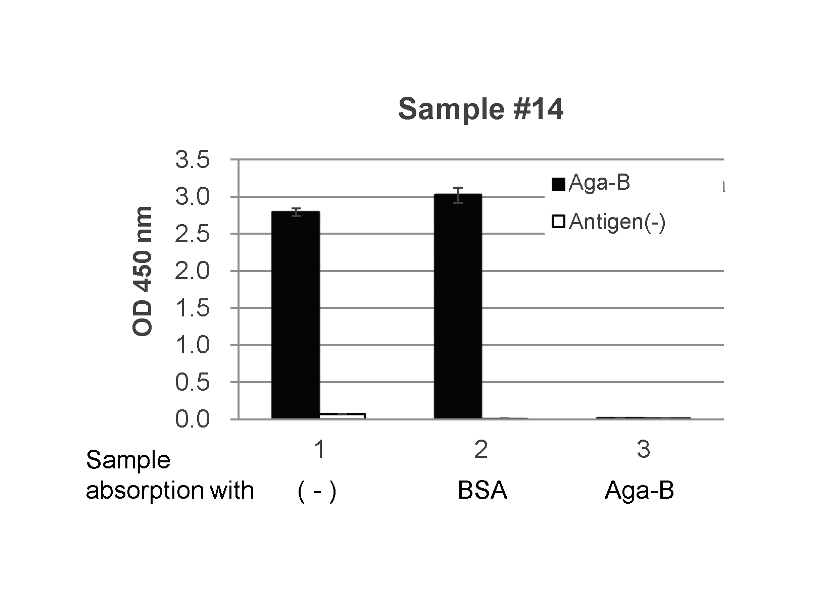

Supplement: S5 Fig — (TIF) [file pone.0128351.s005.tif]
