# Supplementary material for: Rapid Immunochromatographic Detection of Serum Anti-α-Galactosidase A Antibodies in Fabry Patients after Enzyme Replacement Therapy
Source: PLoS One. 2015 Jun 17;10(6):e0128351. doi: 10.1371/journal.pone.0128351 (PMC4470989; doi:10.1371/journal.pone.0128351)
Supplement: S1 Table — (DOCX) [file pone.0128351.s006.docx]

**S1 Table.** The average values of Anti GLA Antibody in health control

| **Sample No.** | **ELISA** | | | | | | **IC^3)^** | |
| --- | --- | --- | --- | --- | --- | --- | --- | --- |
|  | Aga-A^1)^ | | Aga-B^2)^ | | Ag: (-) | | Aga-A | Aga-B |
|  | OD^4)^ | SD | OD | SD | OD | SD | Score^5)^ | |
| 1 | 0.029 | 0.002 | 0.028 | 0.001 | 0.016 | 0.002 | 0 | 0 |
| 2 | 0.015 | 0.002 | 0.012 | 0.003 | 0.019 | 0.003 | 0 | 0 |
| 3 | 0.050 | 0.004 | 0.055 | 0.005 | 0.077 | 0.005 | 0 | 0 |
| 4 | 0.027 | 0.004 | 0.032 | 0.005 | 0.030 | 0.004 | 0 | 0 |
| 5 | 0.008 | 0.002 | 0.008 | 0.001 | 0.008 | 0.002 | 0 | 0 |
| 6 | 0.019 | 0.003 | 0.021 | 0.001 | 0.022 | 0.003 | 0 | 0 |
| 7 | 0.078 | 0.003 | 0.090 | 0.009 | 0.237 | 0.011 | 0 | 0 |
| 8 | 0.018 | 0.002 | 0.028 | 0.005 | 0.019 | 0.002 | 0 | 0 |
| 9 | 0.066 | 0.011 | 0.062 | 0.003 | 0.049 | 0.009 | 0 | 0 |
| 10 | 0.019 | 0.002 | 0.019 | 0.001 | 0.019 | 0.002 | 0 | 0 |
| 11 | 0.012 | 0.002 | 0.015 | 0.001 | 0.017 | 0.003 | 0 | 0 |
| 12 | 0.070 | 0.003 | 0.081 | 0.006 | 0.077 | 0.006 | 0 | 0 |
| 13 | 0.039 | 0.001 | 0.038 | 0.001 | 0.039 | 0.001 | 0 | 0 |
| 14 | 0.018 | 0.001 | 0.023 | 0.001 | 0.016 | 0.004 | 0 | 0 |
| 15 | 0.077 | 0.002 | 0.094 | 0.005 | 0.086 | 0.003 | 0 | 0 |
| 16 | 0.025 | 0.004 | 0.024 | 0.002 | 0.029 | 0.004 | 0 | 0 |
| 17 | 0.038 | 0.003 | 0.034 | 0.001 | 0.075 | 0.001 | 0 | 0 |
| 18 | 0.020 | 0.002 | 0.021 | 0.002 | 0.033 | 0.001 | 0 | 0 |
| 19 | 0.057 | 0.003 | 0.061 | 0.001 | 0.057 | 0.002 | 0 | 0 |
| 20 | 0.019 | 0.001 | 0.021 | 0.005 | 0.019 | 0.005 | 0 | 0 |

^1)^Aga-A; agalsidase alpha for antigen, ^2)^Aga-B; agalsidase beta, ^3)^IC; Immunochromatography, ^4)^OD; optical density at 450nm, ^5)^Score: 8 scale-values of line density by visual measurement.
