# Supplementary material for: Rapid Immunochromatographic Detection of Serum Anti-α-Galactosidase A Antibodies in Fabry Patients after Enzyme Replacement Therapy
Source: PLoS One. 2015 Jun 17;10(6):e0128351. doi: 10.1371/journal.pone.0128351 (PMC4470989; doi:10.1371/journal.pone.0128351)
Supplement: S2 Table — The average values of anti-GLA antibodies in healthy controls. (DOCX) [file pone.0128351.s007.docx]

**S2 Table**. Specificity of Aga-A IC or Aga-B IC for serum or plasma anti-GLAs antibodies in Fabry Patients

| **Sample No.** | **Serum** | **IC with rGLAs**  **(Score)** | | **IC with other enzymes related for lysosomal storage diseases** | | | |
| --- | --- | --- | --- | --- | --- | --- | --- |
|  |  | Aga-B | Aga-A | acid-α-glucosidase | α-L-iduronidase | iduronate-2-sulphatase | modified Naga^1)^ |
| 27 | Serum | 8 | 8 | 0 | 0 | 0 | 0 |
| 28 | Serum | 5 | 1 | 0 | 0 | 0 | 0 |
| 29 | Serum | 0 | 0 | 0 | 0 | 0 | 0 |

^1)^modified Naga; modified α-N-acetylgalactosaminidase (refer to reference 13)
