# Supplementary material for: Rapid Immunochromatographic Detection of Serum Anti-α-Galactosidase A Antibodies in Fabry Patients after Enzyme Replacement Therapy
Source: PLoS One. 2015 Jun 17;10(6):e0128351. doi: 10.1371/journal.pone.0128351 (PMC4470989; doi:10.1371/journal.pone.0128351)
Supplement: S3 Table — (DOCX) [file pone.0128351.s008.docx]

**S3 Table.** Specificity of ELISA with or without pre-absorption of BSA and/or Aga-A in samples from #2 and 5

| **No.** | **Absorp.^1)^** | | **ELISA: Aga-A (OD450 nm)** | | | | | | **ELISA: Ag (-) (OD450 nm)** | | | | | |
| --- | --- | --- | --- | --- | --- | --- | --- | --- | --- | --- | --- | --- | --- | --- |
|  |  | | 1 | 2 | 3 | Aver.^2)^ | S.D. | %C.V. | 1 | 2 | 3 | Aver. | S.D. | %C.V. |
| 5 | 1 | ( - ) | 0.174 | 0.175 | 0.181 | 0.177 | 0.004 | 2.143 | 0.365 | 0.386 | 0.396 | 0.382 | 0.016 | 4.138 |
|  | 2 | + BSA | 0.180 | 0.163 | 0.160 | 0.167 | 0.011 | 6.446 | 0.217 | 0.220 | 0.231 | 0.223 | 0.007 | 3.310 |
|  | 3 | BSA + Aga-A | 0.148 | 0.155 | 0.149 | 0.150 | 0.004 | 2.518 | 0.192 | 0.188 | 0.184 | 0.188 | 0.004 | 2.124 |
| 2 | 1 | ( - ) | 2.961 | 2.868 | 2.944 | 2.924 | 0.050 | 1.693 | 0.052 | 0.057 | 0.062 | 0.057 | 0.005 | 8.772 |
|  | 2 | BSA | 2.736 | 2.805 | 2.638 | 2.726 | 0.084 | 3.078 | 0.012 | 0.012 | 0.012 | 0.012 | 0.000 | 0.000 |
|  | 3 | BSA + Aga-A | 0.057 | 0.061 | 0.060 | 0.059 | 0.002 | 3.528 | 0.017 | 0.014 | 0.013 | 0.015 | 0.002 | 13.878 |

^1)^Absorp.; Absorption methods at 450 nm, ^2)^Aver.; Average
