# Supplementary material for: Rapid Immunochromatographic Detection of Serum Anti-α-Galactosidase A Antibodies in Fabry Patients after Enzyme Replacement Therapy
Source: PLoS One. 2015 Jun 17;10(6):e0128351. doi: 10.1371/journal.pone.0128351 (PMC4470989; doi:10.1371/journal.pone.0128351)
Supplement: S4 Table — (DOCX) [file pone.0128351.s009.docx]

| **No.** | **Absorp.** | | **ELISA: Aga-B (OD450 nm)** | | | | | | **ELISA: Ag (-) blank (OD450 nm)** | | | | | |
| --- | --- | --- | --- | --- | --- | --- | --- | --- | --- | --- | --- | --- | --- | --- |
|  |  | | 1 | 2 | 3 | Aver. | S.D. | %C.V. | 1 | 2 | 3 | Aver. | S.D. | %C.V. |
| 19 | 1 | ( - ) | 0.071 | 0.074 | 0.083 | 0.076 | 0.006 | 8.217 | 0.521 | 0.513 | 0.510 | 0.515 | 0.006 | 1.104 |
|  | 2 | BSA | 0.071 | 0.073 | 0.144 | 0.096 | 0.042 | 43.465 | 0.029 | 0.032 | 0.034 | 0.032 | 0.003 | 7.864 |
|  | 3 | BSA + Aga-B | 0.046 | 0.044 | 0.042 | 0.044 | 0.002 | 4.545 | 0.034 | 0.031 | 0.024 | 0.029 | 0.005 | 17.494 |
| 14 | 1 | ( - ) | 2.827 | 2.810 | 2.734 | 2.790 | 0.050 | 1.775 | 0.073 | 0.068 | 0.069 | 0.070 | 0.003 | 3.762 |
|  | 2 | BSA | 3.134 | 2.987 | 2.939 | 3.020 | 0.102 | 3.365 | 0.006 | 0.009 | 0.008 | 0.008 | 0.002 | 19.094 |
|  | 3 | BSA + Aga-B | 0.017 | 0.018 | 0.019 | 0.018 | 0.001 | 5.556 | 0.010 | 0.012 | 0.010 | 0.010 | 0.001 | 11.170 |

**S4 Table.** Specificity of ELISA with or without pre-absorption of BSA and/or Aga-B in samples from #14 and 19
